# Supplementary material for: Prevalence of Individuals at Clinical High-Risk of Psychosis in the General Population and Clinical Samples: Systematic Review and Meta-Analysis
Source: Brain Sci. 2021 Nov 20;11(11):1544. doi: 10.3390/brainsci11111544 (PMC8615691; doi:10.3390/brainsci11111544)
Supplement: Supplementary file 1 [file brainsci-11-01544-s001.zip › brainsci-1443994-supplementary.pdf]

SUPPLEMENTARY MATERIAL

Table S1, p2-3. PRISMA statement and checklist
Table S2, p4-5. MOOSE checklist
Table S3, p6: Risk of bias assessment using modified Newcastle Ottawa Scale for cross-sectional and cohort studies.
Table S4, p7-8: Characteristics of the included studies conducted in the general population.
Table S5, p9-11: Characteristics of the included studies conducted in clinical samples
Table S6, p12: Meta-regression analyses.
Methods S1, p13: Gold-standard CHR-P assessments.
Methods S2, p14: Pre-screening CHR-P instruments
Results S1, p15: Leave-one study out sensitivity analyses
Discussion S1, p16: CAARMS and SIPS
Figure S1, p17: Meta-funnel CHR-P individuals in the general population.
Figure S2, p18: Forest plot % CHR-P individuals in clinical populations initially negative according to pre-screening instruments.
Figure S3, p19: Meta-funnel CHR-P individuals in clinical samples.
Figure S4, p20: Forest plot leave-one study out sensitivity analyses prevalence CHR-P individuals in the general population
Figure S5, p21: Forest plot leave-one study out sensitivity analyses prevalence CHR-P individuals in clinical samples

This supplementary material has been provided by the authors to give readers additional information about their work..

Table S1: PRISMA statement and checklist

Section/topic	#	Checklist item	Section
**TITLE**			
Title	1	Identify the report as a systematic review, meta-analysis, or both.	Title
**ABSTRACT**			
Structured summary	2	Provide a structured summary including, as applicable: background; objectives; data sources; study eligibility criteria, participants, and interventions; study appraisal and synthesis methods; results; limitations; conclusions and implications of key findings; systematic review registration number.	Abstract
**INTRODUCTION**			
Rationale	3	Describe the rationale for the review in the context of what is already known.	Introduction
Objectives	4	Provide an explicit statement of questions being addressed with reference to participants, interventions, comparisons, outcomes, and study design (PICOS).	Introduction
**METHODS**			
Protocol and registration	5	Indicate if a review protocol exists, if and where it can be accessed (e.g., Web address), and, if available, provide registration information including registration number.	Methods
Eligibility criteria	6	Specify study characteristics (e.g., PICOS, length of follow-up) and report characteristics (e.g., years considered, language, publication status) used as criteria for eligibility, giving rationale.	Methods, supplementary
Information sources	7	Describe all information sources (e.g., databases with dates of coverage, contact with study authors to identify additional studies) in the search and date last searched.	Methods
Search	8	Present full electronic search strategy for at least one database, including any limits used, such that it could be repeated.	Methods
Study selection	9	State the process for selecting studies (i.e., screening, eligibility, included in systematic review, and, if applicable, included in the meta-analysis).	Methods
Data collection process	10	Describe method of data extraction from reports (e.g., piloted forms, independently, in duplicate) and any processes for obtaining and confirming data from investigators.	Methods
Data items	11	List and define all variables for which data were sought (e.g., PICOS, funding sources) and any assumptions and simplifications made.	Methods
Risk of bias in individual studies	12	Describe methods used for assessing risk of bias of individual studies (including specification of whether this was done at the study or outcome level), and how this information is to be used in any data synthesis.	Methods, supplementary

Summary measures	13	State the principal summary measures	Methods
Risk of bias across studies	15	Specify any assessment of risk of bias (i.e. Newcastle-Ottawa Scale (NOS), that may affect the cumulative evidence.	Methods
Additional analyses	16	Describe methods of additional analyses (e.g., sensitivity or subgroup analyses, meta-regression), if done, indicating which were pre-specified.	Methods
**RESULTS**			
Study selection	17	Give numbers of studies screened, assessed for eligibility, and included in the review, with reasons for exclusions at each stage, ideally with a flow diagram.	Results
Study characteristics	18	For each study, present characteristics for which data were extracted (e.g., study size, PICOS, follow-up period) and provide the citations.	Results, supplementary
Risk of bias within studies	19	Present data on risk of bias of each study and, if available, any outcome level assessment (see item 12).	Results
Results of individual studies	20	For all outcomes considered (benefits or harms), present, for each study a summary data for each intervention group	Results, supplementary
Synthesis of results	21	Present results of study analyzed	Results
Risk of bias across studies	22	Present results of any assessment of risk of bias across studies (see Item 15).	Results, supplementary
Additional analysis	23	Give results of additional analyses, if done (e.g., sensitivity or subgroup analyses, meta-regression [see Item 16]).	Results, supplementary
**DISCUSSION**			
Summary of evidence	24	Summarize the main findings including the strength of evidence for each main outcome; consider their relevance to key groups (e.g., healthcare providers, users, and policy makers).	Discussion
Limitations	25	Discuss limitations at study and outcome level (e.g., risk of bias), and at review-level (e.g., incomplete retrieval of identified research, reporting bias).	Discussion
Conclusions	26	Provide a general interpretation of the results in the context of other evidence, and implications for future research.	Discussion
**FUNDING**			
Funding	27	Describe sources of funding for the systematic review and other support (e.g., supply of data); role of funders for the systematic review.	Funding

Table S2: MOOSE checklist

Criteria		Brief description of how the criteria were handled in the meta-analysis
**Reporting of background should include**		
√	Problem definition	No meta-analysis has evaluated the presence of individuals fulfilling CHR-P criteria in the general population and clinical samples.
√	Hypothesis statement	We hypothesized that the prevalence would be higher in clinical samples.
√	Description of study outcomes	The primary outcomes are the proportions of CHR-P individuals in the general population and clinical samples.
√	Type of exposure or intervention used	We did not limit our search according to exposure or intervention used.
√	Type of study designs used	Original individual studies were included. Reviews, clinical cases and conference proceedings were excluded.
√	Study population	Both general population and clinical samples were considered separately, and the presence of CHR-P evaluated.
**Reporting of search strategy should include**		
√	Qualifications of searchers	The credentials of the investigators were detailed.
√	Search strategy, including time period included in the synthesis and keywords	We performed a multi-step literature search using the keywords detailed in the methods section from inception until 21st January 2021.
√	Databases and registries searched	Databases included in the Web of Science, Cochrane Central Register of Reviews, Ovid/PsychINFO, Open Grey and preprint databases were searched.
√	Use of hand searching	We hand-searched bibliographies of retrieved papers for additional references.
√	List of citations located and those excluded, including justifications	Details of the literature search process are outlined in the results section and PRISMA flowchart.
√	Method of addressing articles published in languages other than English	Only articles in English were selected.
√	Method of handling abstracts and unpublished studies	Including criteria regarding design are detailed in the text and above. Authors were contacted and grey literature was searched in Open Grey.
√	Description of any contact with authors	We contacted corresponding authors to request additional data as specified in the main text.
**Reporting of methods should include**		
√	Description of relevance or appropriateness of studies assembled for assessing the	Detailed inclusion and exclusion criteria were described in the methods section.
√	Rationale for the selection and coding of data	Data extracted from each of the studies were relevant to the population characteristics, study design, exposure, outcome, and the possible effect of confounders.
√	Assessment of confounding	Meta-regressions were planned as established in the main text.

√	Assessment of study quality, including blinding of quality assessors; stratification	We adapted the Newcastle-Ottawa Scale for the evaluation of cross-sectional and cohort studies to assess the study quality, in line with previous reviews.
√	Assessment of heterogeneity	Heterogeneity was assessed with the I^2 index.
√	Description of statistical methods in sufficient detail to be replicated	We estimated the proportion of CHR-P individuals in the general population and clinical samples as proportions (95%CI). Random effect meta-analyses were conducted. Heterogeneity among study point estimates was assessed using Q statistics. The proportion of the total variability in the effect size estimates was evaluated with the I^2 index [1]. Sensitivity analyses and meta-regressions were conducted as detailed in the main text.
√	Provision of appropriate tables and graphics	We included tables and graphics to describe our results.
**Reporting of results should include**		
√	Graph summarizing individual study estimates and overall estimate	We have included them in our manuscript.
√	Table giving descriptive information for each study included	We have presented descriptive information for each study in the supplementary material (eTables 4-5).
√	Results of sensitivity testing	We carried out sensitivity analysis to compare a) type of CHR-P interview: studies using the SIPS vs studies using the CAARMS and b) type of assessment: studies using only the gold-standard CHR-P instrument vs those using first a pre-screening instrument and then the gold-standard CHR-P instrument for those individuals testing positive at the pre-screening test. Other sensitivity analyses compared c) studied conducted in school/colleges vs other studies within the general population group and d) forensic samples vs other samples within the clinical samples group.
√	Indication of statistical uncertainty of findings	We reported mean estimates and 95% IC for our outcomes.
**Reporting of discussion should include**		
√	Quantitative assessment of bias	The presence of publication bias in the results was assessed by the Egger´s text and by visually inspecting funnel plots. The use of the "trim and fill" method was planned to correct the effects of any publication bias detected.
√	Justification for exclusion	We excluded reviews, clinical cases or conference proceedings because we consider them inadequate designs to answer our research questions; we excluded studies using only non-established psychometric instruments as we did not consider them valid to establish definite CHR-P designations; we excluded studies in which all the individuals already were suspected to be at CHR-P because they would artificially increase the prevalence.
√	Assessment of quality of included studies	The quality of included studies was reported and discussed.
**Reporting of conclusions should include**		
√	Consideration of alternative explanations for observed results	We discussed other explanations for our findings, considering potential methodological shortcomings.
√	Generalization of the conclusions	We have addressed the generalization of the conclusions in the discussion section.
√	Guidelines for future research	We have suggested possible streams of future development and research in the discussion.
√	Disclosure of funding source	No separate funding was necessary for the undertaking this meta-analysis.

Table S3: Risk of bias assessment using modified Newcastle Ottawa Scale for cross-sectional and cohort studies [2]

Criteria	Maximum Score
Cross-sectional Studies	
Sample representative of target sample (e.g., all eligible or random sample)?	2
Sample size justified and satisfactory?	1
Non-response rate defined, satisfactory, and characteristics of responders/non-responders compared?	1
Ascertainment of exposure (i.e., menstrual cycle) valid and/or well-described?	1
Assessment of outcome with a robust tool and/or record linkage?	2
Outcome per group reported appropriately?	1
Cohort Studies	
Cohort representative (e.g., total population or random sample, selected group)?	1
Method used to ascertain exposure is robust?	1
Are groups matched or adjusted for confounding factors?	2
Assessment of outcome was blind to exposure status or used record linkage, were robust tools used?	2
Follow-up period was sufficiently long for outcomes to occur?	1
Loss to follow-up rate reported, low (<30%), and similar in exposed and non-exposed?	1

Table S4: Characteristics of the included studies conducted in the general population

Author and year	Country	General population type (age mean or range)	Assessment type (pre-screening tool, if applicable)	Gold-standard CHR-P assessment instrument	Assessed sample (sample meeting CHR-P criteria)	CHR-P subgroups[a]	Age: mean, SD (range) CHR-P	Sex: % female CHR-P	NOS
Wang, 2015 [3]	China	College students (18.8)	Pre-screening (PQ-16) then gold-standard CHR-P assessment	SIPS/SOPS	2800 (32)	100% APS, 21.8% GRD	N.a.	40.6	7
Veijola, 2013 [4]	Finland	General population (22.5, 19-25)	Pre-screening (PROD-screen) then gold-standard CHR-P assessment	SIPS/SOPS	9156 (29)	N.a.	22.2 (19-25)	59	5
Svirskis, 2005 **[5]**	Finland	General population (N.a.)	Gold-standard CHR-P assessment only	SIPS/SOPS	34 (3)	N.a.	N.a.	N.a.	6
Kelleher, 2012 [6]	Ireland	School students (11-13)	Pre-screening (APSS) then gold-standard CHR-P assessment	SIPS/SOPS, CAARMS	212 (2)	94.7% APS, 42.1% BLIPS	(11.0-13)	N.a.	8
Koren, 2016 [7]	Israel	School students (13.4, 13-16)	Pre-screening (PQ) then gold-standard CHR-P assessment	SIPS/SOPS	100 (12)	100% APS	13.9, 0.7 (13-16)	83.3	7
Chung, 2013 [8]	Korea	School students (13-15)	Pre-screening (K-YSR) then gold-standard CHR-P assessment	CAARMS	1002 (13)	N.a.	(13-15)	N.a.	7
Razali, 2015 [9]	Malaysia	General population (20.3, 12-30)	Pre-screening (SQ) then gold-standard CHR-P assessment	CAARMS	660 (9)	N.a.	(12.0-30)	28.4[b]	7
Kim, 2018 [10]	Republic of Korea	College students (18-23)	Pre-screening (PQ-16) then gold-standard CHR-P assessment	CAARMS	2246 (17)	N.a.	(18-23)	50.2[c]	7
Schultze-Lutter, 2020 [11]	Switzerland	General population (28.8, 8-40)	Gold-standard CHR-P assessment only	SIPS/SOPS, SPI-A, SPI-CY	2916 (38)	52.6% APS, 2.6% BIPS, 55.3% COGDIS	(8.0-40)	45.8[c]	6

Sullivan, 2020 [12]	Switzerland	General population (24)	Gold-standard CHR-P assessment only	SIPS/SOPS, CAARMS	3866 (36)	N.a.	24.0, 0.8	60.8	4
McDonald, 2019 [13]	UK	General population (16-35)	Pre-screening (PQ-16) then gold-standard CHR-P assessment	CAARMS, SPI-A	2297 (87)	N.a.	22.0, 4.0 (16-35)	76.2	6
Woods, 2010 [14]	USA	General population (25, 22-28)	Gold-standard CHR-P assessment only	SIPS/SOPS	30 (1)	100% APS	(22-28)	N.a.	4
Kim 2020 [15]	Korea	College students (21.8, 18-30)	Pre-screening (PQ-16) then gold-standard CHR-P assessment	CAARMS	1749 (12)	100% APS	(18-30)	74.3	7

[a]>100% because participants may fulfill more than one criteria; [b]Participants were assessed with the CAARMS in a second step. [c]Data obtained from CHR-P and non-CHR-P individuals in the study;

APS: Attenuated Psychosis Symptoms; APSS: Adolescent Psychotic-Like Symptom Screener; BIPS: Brief Intermittent Psychotic Symptoms; BLIPS: Brief Limited Intermittent Psychotic Symptoms; CAARMS: Comprehensive Assessment of At-Risk Mental States; CHR-P: Clinical high risk of psychosis; GRD: Genetic risk and deterioration syndrome; K-YSR: Youth Self-Report; N.a.: not available; NOS: Newcastle-Ottawa Scale; PQ: Prodromal Questionnaire; SIPS: Structured Interview for Prodromal Syndromes; SOPS: Scale of Psychosis-risk Symptoms; SPI: Schizophrenia Proneness Instrument; SPI-A: Schizophrenia Proneness Instrument–Adult; SQ: Screening questionnaire; UK: United Kingdom; USA: United States of America.

Table S5: Characteristics of the included studies conducted in clinical samples

Author and year	Country	Clinical sample type (age mean or range)	Assessment type (pre-screening tool, if applicable)	Gold-standard CHR-P assessment instrument	Total sample assessed (sample meeting CHR-P criteria)	CHR-P subgroups[a]	Age: mean, SD (range) CHR-P	Sex: % female CHR-P	NOS
Hazan, 2019 [16]	Australia	Help-seeking young people (18.3, 12-25)	Gold-standard CHR-P assessment only	CAARMS	465 (173)	100% APS	18.3, 3.3 (12-25)[b]	68.4[b]	5
Yung, 2008 [17]	Australia	Help-seeking young people (18.1, 15-24)	Gold-standard CHR-P assessment only	CAARMS	292 (119)	93.3% APS, 10.9% GRD	(15-24)[b]	51[b]	6
Zhang, 2015 [18]	China	Help-seeking young people (27.1, 15-45)	Pre-screening (PQ-B) then gold-standard CHR-P assessment	SIPS/SOPS	2101 (91)	70.3 % APS, 3.3% BLIPS, 27.5% GRD	25.9, 7.5 (15-45)	49.5	8
Xu, 2018 [19]	China	Help-seeking young people (23.1, 15-45)	Pre-screening (PS-R) then gold-standard CHR-P assessment	SIPS/SOPS	566 (112)	92.0% APS, 5.4% BIPS, 7.2% GRD	22.0, 6.5 (15-45)	58.9	6
Lindgren, 2010 [20]	Finland	Help-seeking young people (16.6, 15-18)	Pre-screening (PQ) then gold-standard CHR-P assessment	SIPS/SOPS	189 (62)	96.8% APS. 1.6% BLIPS, 4.8% GRD	16.6, 0.9 (15-18)	79.0	8
Manninen, 2014 [21]	Finland	Adolescents with disruptive behaviors in reform school (15-18)	Gold-standard CHR-P assessment only	SIPS/SOPS	52 (7)	100% APS	(15-18)	28.6	6
Kaligis, 2018 [22]	Indonesia	Help-seeking adolescents (14, 10-18)	Gold-standard CHR-P assessment only	SIPS/SOPS	50 (17)	N.a.	(10-18)	58	4
Flynn 2012 [23]	Ireland	Youth offenders (18.2, 16-20)	Gold-standard CHR-P assessment only	CAARMS	171 (39)	74.4% APS, 7.7% BLIPS, 35.9% GRD	18.1, 1.4 (16-20)	0	7
Koren, 2013 [24]	Israel	Help-seeking adolescents (15.9, 13-18)	Pre-screening (PQ) then gold-standard CHR-P assessment	SIPS/SOPS	82 (28)	100% APS	15.9, 1.4 (14-18)	36.8	6
Comparelli, 2010 [25]	Italy	Help-seeking young people (23.0, 15-30)	Gold-standard CHR-P assessment only	SIPS/SOPS	128 (26)	N.a.	(15-30)	64.1[b]	7

Masillo, 2018 [26]	Italy	Help-seeking young people (17.4, 12-35)	Pre-screening (PQ-92) then gold-standard CHR-P assessment	SIPS/SOPS	338 (64)	100% APS	(12.0-35)	48.4	7
Lo Cascio 2017 [27]	Italy	Help-seeking young people (15.2, 12-21)	Gold-standard CHR-P assessment only	SIPS/SOPS	237 (39)	100% APS	15.3, 1.8 (12.0-21)	48.7	7
Raballo, 2018 [28]	Italy	Help-seeking adolescents (15.5, 14-18)	Gold-standard CHR-P assessment only	SIPS/SOPS	96 (23)	95.7% APS, 4.3% BLIPS	15.6, 1.2 (14-18)	52.2	6
Raballo, 2016 [29]	Italy	Help-seeking young people (20.2, 14-25)	Gold-standard CHR-P assessment only	SIPS/SOPS	47 (29)	96.6% APS, 3.4% BLIPS, 27.6% COGDIS	20.3, 2.8 (14-25)	25.8	6
Kobayashi, 2008 [30]	Japan	Help-seeking young people (23.6, 16-30)	Pre-screening (PS-R) then gold-standard CHR-P assessment	SIPS/SOPS	115 (19)	N.a	(16-30)	59.7	5
Ising, 2012 [31]	Netherlands	Help-seeking young people (26.2, 18-34)	Pre-screening (PQ-16) then gold-standard CHR-P assessment	CAARMS	3671 (156)	N.a.	(18-35)	68.5	7
Jarrett, 2012 [32]	UK	Male prisoners (28.7, 21-40)	Pre-screening (PQ-B) then gold-standard CHR-P assessment	CAARMS	750 (38)	N.a.	27.9, 6.1 (21-40)	0	7
Salazar de Pablo, 2020 [33]	USA	Help-seeking adolescents (15.4, 12-18)	Gold-standard CHR-P assessment only	SIPS/SOPS	248 (65)	100% APS	15.5, 1.3 (12.0-18)	75.4	7
Thompson2020 [34]	USA	Help-seeking college students (21.8, >18)	Pre-screening. (PRIME-Screen) then gold-standard CHR-P assessment	CAARMS	510 (17)	N.a.	21.1, 3.1 (>18)	54.7	5
Tsuji, 2019 [35]	USA	Help-seeking young people (12-25)	Gold-standard CHR-P assessment only	SIPS/SOPS	147 (52)	N.a.	(12.0-25)	61.9	5
Wilson 2020 [36]	USA	Individuals with autism (14.7, 12-18)	Gold-standard CHR-P assessment only	SIPS/SOPS	21 (0)	D.n.a.	N.a.	14	5

Yung 2005 [37]	Australia	Help-seeking young people (15-24)	Gold-standard CHR-P assessment only	CAARMS	150 (43)	N.a.	(15-24)	N.a.	6

[a] >100% because participant may fulfill more than one criteria; [b]Data obtained from study sample with both CHR-P and non CHR-P individuals;
APS: Attenuated Psychosis Symptoms; BIPS: Brief Intermittent Psychotic Symptoms; BLIPS: Brief Limited Intermittent Psychotic Symptoms; CAARMS: Comprehensive Assessment of At-Risk Mental States; CHR-P: Clinical high risk of psychosis; GRD: Genetic risk and deterioration syndrome; N.a.: not available; NOS: Newcastle-Ottawa Scale; PQ: Prodromal Questionnaire; PQ-B: Prodromal Questionnaire-Brief; PS: Prime Screen; PS-R: Prime Screen- Revised; SIPS: Structured Interview for Prodromal Syndromes; SOPS: Scale of Psychosis-risk Symptoms; UK: United Kingdom; USA: United States of America; Y-PARQ: Youth Psychosis At-Risk Questionnaire.

Table S6: Meta-regression analyses

Outcome	Moderator	No. of Studies	β coefficient	SE	95% CI		Z-Value	P value
**General populations**	Age	8	-2.865	1.802	-6.398	0.667	-1.590	0.112
	Sex	8	0.045	0.022	0.002	0.089	2.0450	**0.041**
	NOS	13	0.252	0.329	-0.394	0.897	0.764	0.445
	Continent	13	0.639	0.874	-1.074	2.351	0.731	0.465
**Clinical samples**	Age	20	-0.160	0.036	-0.231	-0.089	-4.405	**<0.001**
	Sex	21	0.008	0.012	-0.015	0.032	0.690	0.490
	NOS	22	-0.226	0.210	-0.637	0.185	-1.077	0.282
	Continent	22	0.127	0.474	-0.802	1.057	0.269	0.788

Methods S1: Gold-standard CHR-P assessments

The CHR-P state comprises the Ultra High Risk state and/or the Basic Symptoms including these instruments (modified from [38]):

The following instruments are considered as validated to define the UHR state: Comprehensive Assessment of At-Risk Mental States (CAARMS [37]) and Structured Interview for Psychosis-risk Syndromes (SIPS [39,40]) and Early Recognition Inventory (ERIraos [41]). Furthermore, before the development of these instruments, the CHR-P state was defined through the Positive and Negative Syndrome Scale (PANSS [42]), Brief Psychiatric Rating Scale (BPRS [43]).

The following instruments are considered as validated to define the BS criteria [38]: Bonn Scale for the Assessment of Basic Symptoms (BSABS [44]), Basel Screening Instrument for Psychosis (BSIP [45]), and Schizophrenia Proneness Instrument [46] - Adult (SPI-A) and Child and Youth (SPI-CY) version-.

As established in previous BS guidelines [47], individuals with Cognitive Disturbances (COGDIS) but not Cognitive-Perceptive Basic Symptoms (COPER) were accepted.

These instruments were all considered in the current meta-analysis as gold-standard CHR-P assessment instruments.

Methods S2: Pre-screening CHR-P instruments

Pre-screening CHR-P instruments employed by studies included in the current review:

- Prodromal Questionnaire (PQ) [48], PQ- Brief version (PQ-B) [49], 16-item PQ (PQ-16) [31], 92-item PQ (PQ-92) [26]: 12 studies (57.1%)
- PRIME Screen (PS) [50] or PRIME Screen –Revised (PS-R) [30]: 4 studies (19.2%)
- PROD-screen [51]: 2 studies (9.5%)
- Adolescent Psychotic-Like Symptom Screener (APSS) [52]: 1 study (4.8%)
- Youth Self-Report (K-YSR)25 [53]: 1 study (4.8%)
- Screening questionnaire (SQ) [9]: 1 study (4.8%)
- Youth Psychosis At-Risk Questionnaire (Y-PARQ) [54]: 1 study (4.8%)

The cut-off scores employed across these instruments to ascertain a CHR-P state [55] were operationalized by each single study with no restriction for inclusion in the current meta-analysis. However, as indicated in the methods, we only included studies which subsequently employed the gold-standard CHR-P assessment instruments to validate the pre-screening assessment. The gold-standard CHR-P instruments are detailed in eMethods 1.

Results S1: Leave-one study out sensitivity analyses

When we repeated the meta-analysis of the CHR-P prevalence in the general population leaving one study out each time, the new prevalence ranged from 1.4% (95%CI 0.8-2.4%) [7] to 1.9% (95%CI 1.2-3.1%) [4]. In both cases, the 95%CI and the mean±SD included the pooled estimate (see eFigure 4), indicating no substantial effect of single studies on the robustness of the findings.

When we repeated the meta-analysis of the CHR-P prevalence in the clinical samples leaving one study out each time, the new prevalence ranged from 17.9% (95%CI 11.9-26.0%) [29] to 20.7% [31,34] (95%CI 13.9-29.7% [34] and 95%CI 14.6-28.5% [31] respectively). In both cases, the 95%CI and the mean±SD included the pooled estimate (see eFigure 5), indicating no substantial effect of single studies on the robustness of the findings.

Discussion S1: CAARMS and SIPS

CAARMS and SIPS [38,56] deliver comparable prevalence of CHR-P cases, likely based on their excellent and comparable psychometric performance to discriminate those at risk or not [57]. Although the SIPS has shown a relatively higher sensitivity for the prediction of psychosis than the CAARMS [58], this difference did not influence the prevalence of cases identified.

Figure S1: Meta-funnel CHR-P individuals in the general population

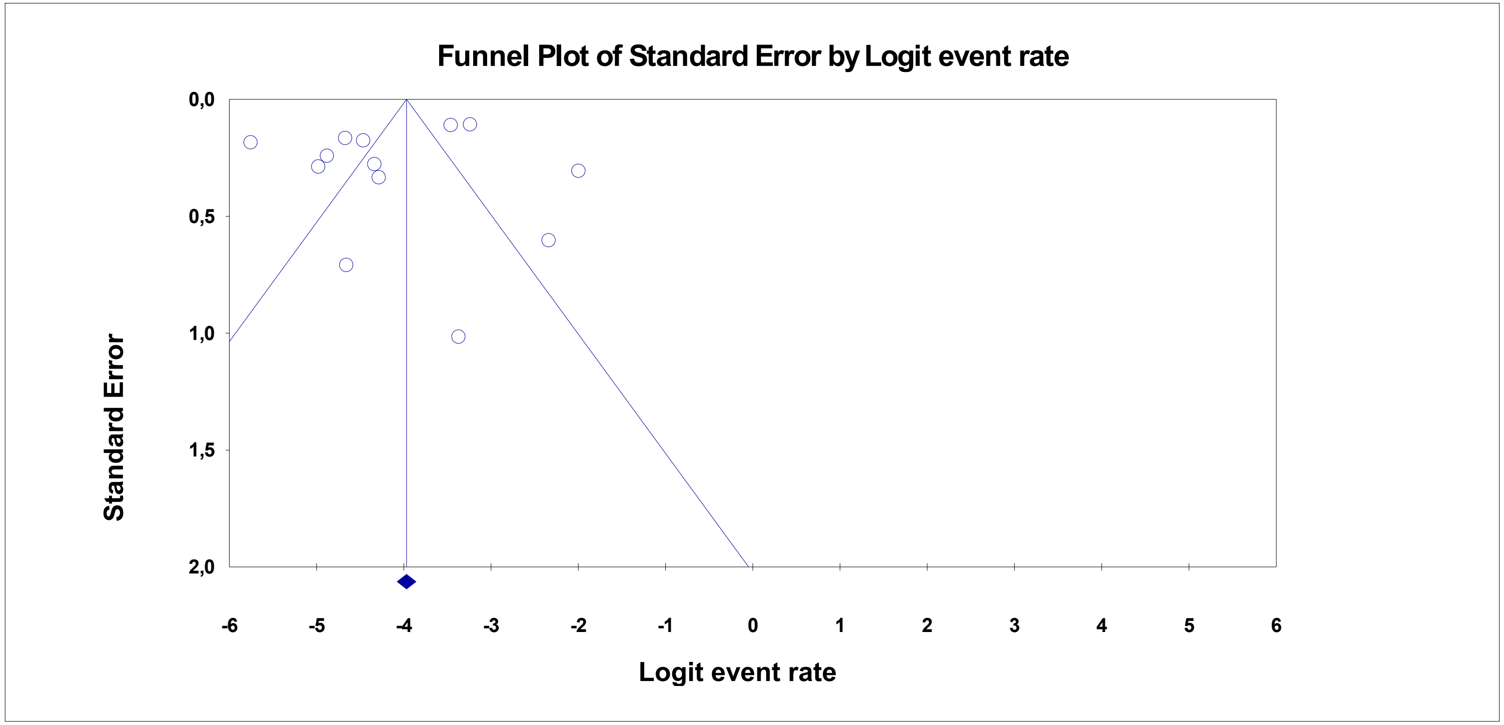

Figure S2: Forest plot % CHR-P individuals in clinical populations initially negative according to pre-screening instruments

Study name	Statistics for each study				
	Event rate	Lower limit	Upper limit	Z-Value	p-Value
Xu 2018	0,125	0,085	0,180	-8,917	0,000
Kobayashi 2008	0,007	0,000	0,104	-3,477	0,001
Ising 2012	0,061	0,032	0,113	-7,935	0,000
Jarrett 2012	0,017	0,002	0,109	-4,043	0,000
	0,056	0,022	0,133	-5,823	0,000

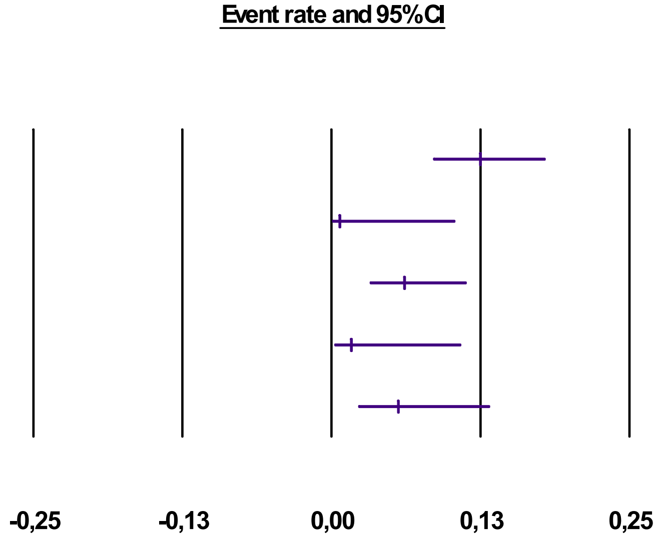

Figure S3: Meta-funnel CHR-P individuals in clinical samples

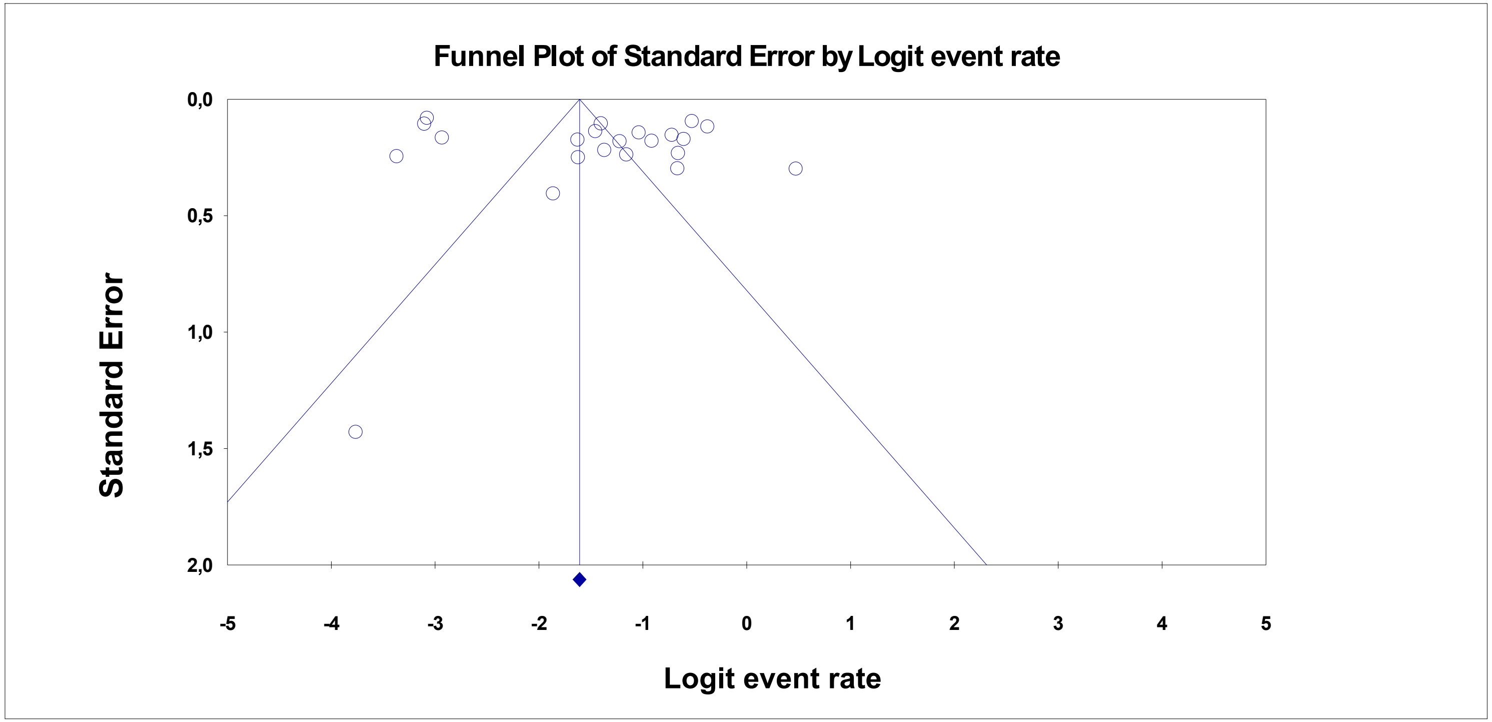

Figure S4: Forest plot leave-one study out sensitivity analyses prevalence CHR-P individuals in the general population

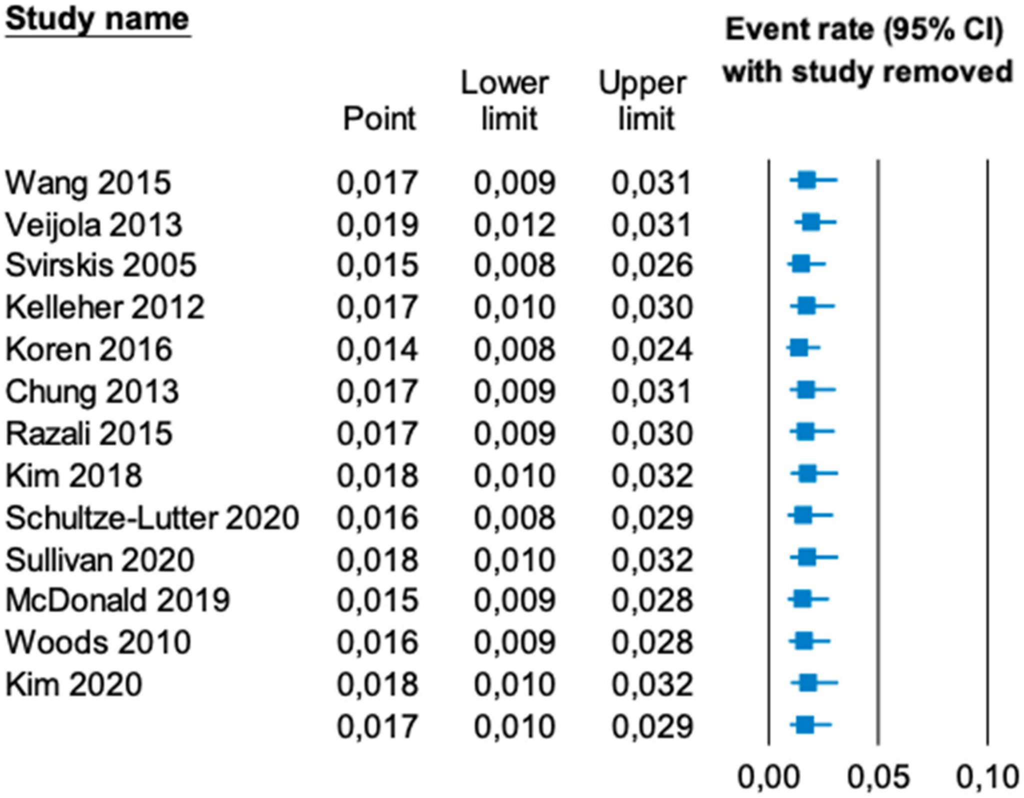

Figure S5: Forest plot leave-one study out sensitivity analyses prevalence CHR-P individuals in clinical samples

Study name	Point	Lower limit	Upper limit
Koren 2013	0,186	0,123	0,272
Hazan 2019	0,185	0,123	0,269
Yung 2008	0,184	0,123	0,267
Zhang 2015	0,206	0,141	0,291
Xu 2018	0,192	0,124	0,283
Lindgren 2010	0,187	0,123	0,273
Manninen 2014	0,195	0,130	0,283
Kaligis 2018	0,187	0,124	0,272
Flynn 2012	0,190	0,126	0,278
Comparelli 2010	0,192	0,127	0,279
Masillo 2018	0,192	0,126	0,282
Lo Cascio 2017	0,194	0,128	0,283
Raballo 2018	0,190	0,126	0,277
Raballo 2016	0,179	0,119	0,260
Kobayashi 2008	0,194	0,128	0,282
Ising 2012	0,207	0,146	0,285
Jarrett 2012	0,204	0,137	0,293
Salazar de Pablo 2020	0,189	0,124	0,277
Tsuji 2019	0,186	0,123	0,271
Wilson 2020	0,199	0,133	0,286
Yung 2005	0,188	0,124	0,275
Thompson 2020	0,207	0,139	0,297
	0,192	0,129	0,277

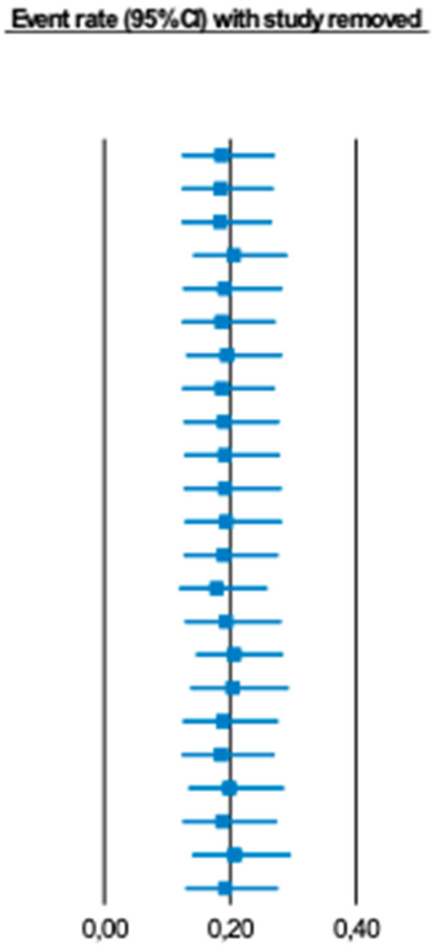

REFERENCES

1. Lipsey, M.; Wilson, D. Practical Meta-analysis. **2000**.

2. Salazar de Pablo, G.; Catalan, A.; Fusar-Poli, P. Clinical Validity of DSM-5 Attenuated Psychosis Syndrome: Advances in Diagnosis, Prognosis, and Treatment. *JAMA Psychiatry* **2019**, doi:10.1001/jamapsychiatry.2019.3561.

3. Wang, L.; Shi, J.; Chen, F.; Yao, Y.; Zhan, C.; Yin, X.; Fang, X.; Wang, H.; Yuan, J.; Zhao, X. Family Perception and 6-Month Symptomatic and Functioning Outcomes in Young Adolescents at Clinical High Risk for Psychosis in a General Population in China. *PLoS One* **2015**, *10*, e0138361, doi:10.1371/journal.pone.0138361.

4. Veijola, J.; Maki, P.; Jaaskelainen, E.; Koivukangas, J.; Moilanen, I.; Taanila, A.; Nordstrom, T.; Hurtig, T.; Kiviniemi, V.; Mukkala, S.; et al. Young people at risk for psychosis: case finding and sample characteristics of the Oulu Brain and Mind Study. *Early Intervention in Psychiatry* **2013**, *7*, 146-154, doi:10.1111/j.1751-7893.2012.00360.x.

5. Svirskis, T.; Korkeila, J.; Heinimaa, M.; Huttunen, J.; Ilonen, T.; Ristkari, T.; McGlashan, T.; Salokangas, R.K.R. Axis-I disorders and vulnerability to psychosis. *Schizophrenia Research* **2005**, *75*, 439-446, doi:10.1016/j.schres.2004.11.002.

6. Kelleher, I.; Murtagh, A.; Molloy, C.; Roddy, S.; Clarke, M.C.; Harley, M.; Cannon, M. Identification and Characterization of Prodromal Risk Syndromes in Young Adolescents in the Community: A Population-Based Clinical Interview Study. *Schizophrenia Bulletin* **2012**, *38*, 239-246, doi:10.1093/schbul/sbr164.

7. Koren, D.; Lacoua, L.; Rothschild-Yakar, L.; Parnas, J. Disturbances of the Basic Self and Prodromal Symptoms Among Young Adolescents From the Community: A Pilot Population-Based Study. *Schizophrenia Bulletin* **2016**, *42*, 1216-1224, doi:10.1093/schbul/sbw010.

8. Chung, Y.-C.; Kang, N.-I.; Im, Y.-J.; Kim, S.-W.; Cho, I.H.; Lee, Y.M.; Kwon, J.S. Validation of the Korean version of the Eppendorf Schizophrenia Inventory as a screening measure to detect adolescents at ultra-high risk for psychosis. *Early Intervention in Psychiatry* **2013**, *7*, 71-79, doi:10.1111/j.1751-7893.2012.00363.x.

9. Razali, S.M.; Abidin, Z.Z.; Othman, Z.; Yassin, M.A.M. Screening for schizophrenia in initial prodromal phase: Detecting the sub-threshold psychosis. *Asian Journal of Psychiatry* **2015**, *16*, 26-31, doi:10.1016/j.ajp.2015.06.011.

10. Kim, S.-W.; Chung, Y.-C.; Kang, Y.-S.; Kim, J.-K.; Jang, J.-E.; Jhon, M.; Lee, J.-Y.; Kim, J.-M.; Shin, I.-S.; Yoon, J.-S. Validation of the Korean version of the 16-Item Prodromal Questionnaire in a Non-Help-Seeking College Population. *Psychiatry Investigation* **2018**, *15*, 111-117, doi:10.30773/pi.2017.04.24.

11. Schultze-Lutter, F.; Schimmelmann, B.G.; Flückiger, R.; Michel, C. Effects of age and sex on clinical high-risk for psychosis in the community. *World J Psychiatry* **2020**, *10*, 101-124, doi:10.5498/wjp.v10.i5.101.

12. Sullivan, S.A.; Kounali, D.; Cannon, M.; David, A.S.; Fletcher, P.C.; Holmans, P.; Jones, H.; Jones, P.B.; Linden, D.E.J.; Lewis, G.; et al. A Population-Based Cohort Study Examining the Incidence and Impact of Psychotic Experiences From Childhood to Adulthood, and Prediction of Psychotic Disorder. *Am J Psychiatry* **2020**, *177*, 308-317, doi:10.1176/appi.ajp.2019.19060654.

13. McDonald, M.; Christoforidou, E.; Van Rijsbergen, N.; Gajwani, R.; Gross, J.; Gumley, A.I.; Lawrie, S.M.; Schwannauer, M.; Schultze-Lutter, F.; Uhlhaas, P.J. Using Online Screening in the General Population to Detect Participants at Clinical High-Risk for Psychosis. *Schizophrenia Bulletin* **2019**, *45*, 600-609, doi:10.1093/schbul/sby069.

14. Woods, S.W.; Walsh, B.C.; Saksa, J.R.; McGlashan, T.H. The case for including Attenuated Psychotic Symptoms Syndrome in DSM-5 as a psychosis risk syndrome. *Schizophrenia Research* **2010**, *123*, 199-207, doi:10.1016/j.schres.2010.08.012.

15. Kim, S.W.; Kim, J.K.; Han, J.H.; Jhon, M.; Kim, J.W.; Lee, J.Y.; Kim, J.M.; Na, H.J.; Kang, Y.S.; Chung, Y.C.; et al. Validation of the Korean Version of the 15-Item Community Assessment of Psychic Experiences in a College Population. *Psychiatry Investig* **2020**, *17*, 306-311, doi:10.30773/pi.2019.0215.

16. Hazan, H.; Spelman, T.; Amminger, G.P.; Hickie, I.; McGorry, P.D.; Phillips, L.J.; Purcell, R.; Wood, S.J.; Yung, A.R.; Nelson, B. The prognostic significance of attenuated psychotic symptoms in help-seeking youth. *Schizophrenia research* **2019**, doi:10.1016/j.schres.2019.10.016.

17. Yung, A.R.; Nelson, B.; Stanford, C.; Simmons, M.B.; Cosgrave, E.M.; Killackey, E.; Phillips, L.J.; Bechdolf, A.; Buckby, J.; McGorry, P.D. Validation of "prodromal" criteria to detect individuals at ultra high risk of psychosis: 2 year follow-up. *Schizophrenia Research* **2008**, *105*, 10-17, doi:10.1016/j.schres.2008.07.012.

18. Zhang, T.; Li, H.; Tang, Y.; Li, H.; Zheng, L.; Guo, Q.; Zhao, S.; Zhuo, K.; Qian, Z.; Wang, L.; et al. Screening schizotypal personality disorder for detection of clinical high risk of psychosis in Chinese mental health services. *Psychiatry Research* **2015**, *228*, 664-670, doi:10.1016/j.psychres.2015.04.049.

19. Xu, L.; Wang, Y.; Cui, H.; Tang, Y.; Wang, J.; Tang, X.; Zhang, B.; Wei, Y.; Zhu, Y.; Jiang, L.; et al. Identification and prediction of clinical high risk of psychosis in Chinese outpatients using two-stage screening. *Schizophrenia Research* **2018**, *202*, 284-290, doi:10.1016/j.schres.2018.06.026.

20. Lindgren, M.; Manninen, M.; Laajasalo, T.; Mustonen, U.; Kalska, H.; Suvisaari, J.; Moilanen, K.; Cannon, T.D.; Huttunen, M.; Therman, S. The relationship between psychotic-like symptoms and neurocognitive performance

in a general adolescent psychiatric sample. *Schizophr Res* **2010**, *123*, 77-85, doi:10.1016/j.schres.2010.07.025.

21. Manninen, M.; Lindgren, M.; Therman, S.; Huttunen, M.; Ebeling, H.; Moilanen, I.; Suvisaari, J. Clinical high-risk state does not predict later psychosis in a delinquent adolescent population. *Early Intervention in Psychiatry* **2014**, *8*, 87-90, doi:10.1111/eip.12045.

22. Kaligis, F.; Marsubrin, R.I.I.; Wiguna, T.; Noorhana, S.W.; Almasyhur, A.F. Translation and validation study of the prodromal questionnaire brief version into Indonesian language. *Asian Journal of Psychiatry* **2018**, *37*, 96-101, doi:10.1016/j.ajp.2018.08.012.

23. Flynn, D.; Smith, D.; Quirke, L.; Monks, S.; Kennedy, H.G. Ultra high risk of psychosis on committal to a young offender prison: an unrecognised opportunity for early intervention. *Bmc Psychiatry* **2012**, *12*, doi:10.1186/1471-244x-12-100.

24. Koren, D.; Reznik, N.; Adres, M.; Scheyer, R.; Apter, A.; Steinberg, T.; Parnas, J. Disturbances of basic self and prodromal symptoms among non-psychotic help-seeking adolescents. *Psychol Med* **2013**, *43*, 1365-1376, doi:10.1017/S0033291712002322.

25. Comparelli, A.; Savoja, V.; Woods, S.W.; Kotzalidis, G.D.; Pucci, D.; Caltagirone, S.S.; Girardi, P.; Conti, L.; Tatarelli, R. Identification of the prodromes of psychosis in a population of psychiatric service users at their first contact: correlations between prodrome-specific and non-specific psychopathology scales and with socio-occupational functioning. *G. Ital. Psicopat* **2010**, *16*, 239-254.

26. Masillo, A.; Brandizzi, M.; Nelson, B.; Lo Cascio, N.; Saba, R.; Lindau, J.F.; Telesforo, L.; Montanaro, D.; D'Alema, M.; Girardi, P.; et al. Youth mental health services in Italy: An achievable dream? *Early Intervention in Psychiatry* **2018**, *12*, 433-443, doi:10.1111/eip.12328.

27. Lo Cascio, N.; Curto, M.; Pasqualetti, P.; Lindau, J.F.; Girardi, N.; Saba, R.; Brandizzi, M.; Monducci, E.; Masillo, A.; Colafrancesco, G.; et al. Impairment in Social Functioning differentiates youth meeting Ultra-High Risk for psychosis criteria from other mental health help-seekers: A validation of the Italian version of the Global Functioning: Social and Global Functioning: Role scales. *Psychiatry Res* **2017**, *253*, 296-302, doi:10.1016/j.psychres.2017.04.008.

28. Raballo, A.; Monducci, E.; Ferrara, M.; Nastro, P.F.; Dario, C.; Grp, R. Developmental vulnerability to psychosis: Selective aggregation of basic self-disturbance in early onset schizophrenia. *Schizophrenia Research* **2018**, *201*, 367-372, doi:10.1016/j.schres.2018.05.012.

29. Raballo, A.; Pappagallo, E.; Dell' Erba, A.; Lo Cascio, N.; Patane, M.; Gebhardt, E.; Boldrini, T.; Terzariol, L.; Angelone, M.; Trisolini, A.; et al. Self-Disorders and Clinical High Risk for Psychosis: An Empirical Study in Help-Seeking Youth Attending Community Mental Health Facilities. *Schizophrenia Bulletin* **2016**, *42*, 926-932, doi:10.1093/schbul/sbv223.

30. Kobayashi, H.; Nemoto, T.; Koshikawa, H.; Osono, Y.; Yamazawa, R.; Murakami, M.; Kashima, H.; Mizuno, M. A self-reported instrument for prodromal symptoms of psychosis: Testing the clinical validity of the PRIME Screen-Revised (PS-R) in a Japanese population. *Schizophrenia Research* **2008**, *106*, 356-362, doi:10.1016/j.schres.2008.08.018.

31. Ising, H.K.; Veling, W.; Loewy, R.L.; Rietveld, M.W.; Rietdijk, J.; Dragt, S.; Klaassen, R.M.; Nieman, D.H.; Wunderink, L.; Linszen, D.H.; et al. The validity of the 16-item version of the Prodromal Questionnaire (PQ-16) to screen for ultra high risk of developing psychosis in the general help-seeking population. *Schizophr Bull* **2012**, *38*, 1288-1296, doi:10.1093/schbul/sbs068.

32. Jarrett, M.; Craig, T.; Parrott, J.; Forrester, A.; Winton-Brown, T.; Maguire, H.; McGuire, P.; Valmaggia, L. Identifying men at ultra high risk of psychosis in a prison population. *Schizophrenia Research* **2012**, *136*, 1-6, doi:10.1016/j.schres.2012.01.025.

33. Salazar de Pablo, G.; Guinart, D.; Cornblatt, B.A.; Auther, A.M.; Carrión, R.E.; Carbon, M.; Jiménez-Fernández, S.; Vernal, D.L.; Walitza, S.; Gerstenberg, M.; et al. DSM-5 Attenuated Psychosis Syndrome in Adolescents Hospitalized With Non-psychotic Psychiatric Disorders. *Front Psychiatry* **2020**, *11*, 568982, doi:10.3389/fpsyt.2020.568982.

34. Thompson, E.; Andorko, N.; Rouhakhtar, P.; Millman, Z.; Sagun, K.; Han, S.; Chibani, D.; Reeves, G.; Herman, B.; Schiffman, J. Psychosis-Spectrum Screening and Assessment within a College Counseling Center: A Pilot Study Exploring Feasibility and Clinical Need. *Journal of College Student Psychotherapy* **2020**, doi:10.1080/87568225.2020.1797604.

35. Tsuji, T.; Phalen, P.; Rouhakhtar, P.R.; Millman, Z.; Bussell, K.; Thompson, E.; Demro, C.; Roemer, C.; Reeves, G.; Schiffman, J. Using the K-SADS psychosis screen to identify people with early psychosis or psychosis risk syndromes. *Clinical Child Psychology and Psychiatry* **2019**, *24*, 809-820, doi:10.1177/1359104519846582.

36. Wilson, C.S.; Anthony, L.; Kenworthy, L.; Fleischman, R.; Demro, C.; Andorko, N.; Chelsea Armour, A.; Schiffman, J. Feasibility of psychosis risk assessment for adolescents diagnosed with autism. *Autism* **2020**, *24*, 834-850, doi:10.1177/1362361320909173.

37. Yung, A.R.; Yuen, H.P.; McGorry, P.D.; Phillips, L.J.; Kelly, D.; Dell'Olio, M.; Francey, S.M.; Cosgrave, E.M.; Killackey, E.; Stanford, C.; et al. Mapping the onset of psychosis: the Comprehensive Assessment of At-Risk Mental States. *Aust N Z J Psychiatry* **2005**, *39*, 964-971, doi:10.1080/j.1440-1614.2005.01714.x.

38. Fusar-Poli, P.; Salazar de Pablo, G.; Correll, C.U.; Meyer-Lindenberg, A.; Millan, M.J.; Borgwardt, S.; Galderisi, S.; Bechdolf, A.; Pfennig, A.; Kessing, L.V.; et al. Prevention of Psychosis: Advances in Detection, Prognosis, and Intervention. *JAMA Psychiatry* **2020**, doi:10.1001/jamapsychiatry.2019.4779.

39. Fusar-Poli, P.; Cappucciati, M.; Rutigliano, G.; Lee, T.Y.; Beverly, Q.; Bonoldi, I.; Lelli, J.; Kaar, S.J.; Gago, E.; Rocchetti, M.; et al. Towards a Standard Psychometric Diagnostic Interview for Subjects at Ultra High Risk of Psychosis: CAARMS versus SIPS. *Psychiatry J* **2016**, *2016*, 7146341, doi:10.1155/2016/7146341.

40. McGlashan T, W.B., Woods S. *The psychosis-risk syndrome: handbook for diagnosis and follow-up.*; Oxford: Oxford University 2010.

41. Haefner, H.; Bechdolf, A.; Klosterkotter, J.; Maurer, K. Early detection and intervention in psychosis. A practice handbook. **2011**.

42. Kay, S.R.; Fiszbein, A.; Opler, L.A. The positive and negative syndrome scale (PANSS) for schizophrenia. *Schizophr Bull* **1987**, *13*, 261-276, doi:10.1093/schbul/13.2.261.

43. Overall, J.; Gorham, D. The Brief Psychiatric Rating Scale (BPRS): recent developments in ascertainment and scaling. *Psychopharmacol Bull* **1988**, *24*, 97-99.

44. Vollmer-Larsen, A.; Handest, P.; Parnas, J. Reliability of measuring anomalous experience: the Bonn Scale for the Assessment of Basic Symptoms. *Psychopathology* **2007**, *40*, 345-348, doi:10.1159/000106311.

45. Riecher-Rössler, A.; Aston, J.; Ventura, J.; Merlo, M.; Borgwardt, S.; Gschwandtner, U.; Stieglitz, R.D. [The Basel Screening Instrument for Psychosis (BSIP): development, structure, reliability and validity]. *Fortschr Neurol Psychiatr* **2008**, *76*, 207-216, doi:10.1055/s-2008-1038155.

46. Fux, L.; Walger, P.; Schimmelmann, B.G.; Schultze-Lutter, F. The Schizophrenia Proneness Instrument, Child and Youth version (SPI-CY): practicability and discriminative validity. *Schizophr Res* **2013**, *146*, 69-78, doi:10.1016/j.schres.2013.02.014.

47. Schultze-Lutter, F.; Michel, C.; Schmidt, S.J.; Schimmelmann, B.G.; Maric, N.P.; Salokangas, R.K.R.; Riecher-Roessler, A.; van der Gaag, M.; Nordentoft, M.; Raballo, A.; et al. EPA guidance on the early detection of clinical high risk states of psychoses. *European Psychiatry* **2015**, *30*, 405-416, doi:10.1016/j.eurpsy.2015.01.010.

48. Loewy, R.L.; Bearden, C.E.; Johnson, J.K.; Raine, A.; Cannon, T.D. The prodromal questionnaire (PQ): preliminary validation of a self-report screening measure for prodromal and psychotic syndromes. *Schizophr Res* **2005**, *79*, 117-125.

49. Loewy, R.L.; Pearson, R.; Vinogradov, S.; Bearden, C.E.; Cannon, T.D. Psychosis risk screening with the Prodromal Questionnaire--brief version (PQ-B). *Schizophr Res* **2011**, *129*, 42-46, doi:10.1016/j.schres.2011.03.029.

50. Miller, T.; Cicchetti, D.; Markovich, P.; McGlashan, T.; Woods, S.W. The SIPS screen: a brief self-report screen to detect the schizophrenia prodrome. *Schizophr. Res.* **2004**, *70*, 78.

51. Heinimaa, M.; Salokangas, R.K.R.; Ristkari, T.; Plathin, M.; Huttunen, J.; Ilonen, T.; McGlashan, T.H. PROD-screen - a screen for prodromal symptoms of psychosis. *International Journal of Methods in Psychiatric Research* **2003**, *12*, 92-104, doi:10.1002/mpr.146.

52. Kelleher, I.; Harley, M.; Murtagh, A.; Cannon, M. Are screening instruments valid for psychotic-like experiences? A validation study of screening questions for psychotic-like experiences using in-depth clinical interview. *Schizophr Bull* **2011**, *37*, 362-369, doi:10.1093/schbul/sbp057.

53. Oh, K.; Hong, K.; Lee, H. Korean-Youth Self Report (K-YSR). **1997**.

54. Ord, L.M.; Myles-Worsley, M.; Blailes, F.; Ngiralmau, H. Screening for prodromal adolescents in an isolated high-risk population. *Schizophr Res* **2004**, *71*, 507-508, doi:10.1016/j.schres.2004.03.014.

55. Schiffman, J.; Ellman, L.M.; Mittal, V.A. Individual Differences and Psychosis-Risk Screening: Practical Suggestions to Improve the Scope and Quality of Early Identification. *Front Psychiatry* **2019**, *10*, 6, doi:10.3389/fpsyt.2019.00006.

56. Woods, S.W.; Bearden, C.E.; Sabb, F.W.; Stone, W.S.; Torous, J.; Cornblatt, B.A.; Perkins, D.O.; Cadenhead, K.S.; Addington, J.; Powers, A.R.; et al. Counterpoint. Early intervention for psychosis risk syndromes: Minimizing risk and maximizing benefit. *Schizophr Res* **2020**, doi:10.1016/j.schres.2020.04.020.

57. Fusar-Poli, P.; Cappucciati, M.; Rutigliano, G.; Schultze-Lutter, F.; Bonoldi, I.; Borgwardt, S.; Riecher-Rossler, A.; Addington, J.; Perkins, D.; Woods, S.W.; et al. At risk or not at risk? A meta-analysis of the prognostic accuracy of psychometric interviews for psychosis prediction. *World Psychiatry* **2015**, *14*, 322-332, doi:10.1002/wps.20250.

58. Oliver, D.; Kotlicka-Antczak, M.; Minichino, A.; Spada, G.; McGuire, P.; Fusar-Poli, P. Meta-analytical prognostic accuracy of the Comprehensive Assessment of at Risk Mental States (CAARMS): The need for refined prediction. *Eur Psychiatry* **2018**, *49*, 62-68, doi:10.1016/j.eurpsy.2017.10.001.
